# Case of Penetrating Wound of the Chest, with a Fractured Rib

**Published:** 1826-09

**Authors:** 

**Affiliations:** the Middlesex Hospital


II. *Case of penetrating Wound of the Chest, with a fractured Rib.* Treated at the MIDDLESEX HOSPITAL, by Mr. BELL.

Michael Harrington, ætatis forty-five, fell from a ladder (Nov. 13th, 1823,) on the spikes of a railing, one of which entered his side: he hung in this situation until lifted off by some workmen. He was immediately brought to the hospital.

There was a wound below the inferior costa of the right scapula, through which the air rushed violently. On introducing the finger into the wound, the direction of which was upwards and forwards, a rib was found to be fractured, and the finger passed easily into the cavity of the chest. The pulse was quick and full; his breathing laborious; and his countenance was dark and livid, and expressive of great anxiety.

He was immediately bled to twenty ounces; the integuments were brought together, and retained by adhesive straps; a compress was put over the wound, and the thorax was swathed with a bandage.—A strong purge of Calomel and Jalap was ordered to be taken immediately.

In the evening, the difficulty of breathing was very great; the pulse was slow and oppressed. He was again bled, to faintness. He lay on his right side, (the one injured.)

Next day, his breathing was easier; there was no emphysema.

He was now ordered to take a Saline Draught, with the Acetate of Ammonia, three times a-day.

On the sixth day, the adhesion of the wound had so far taken place, that the opening into the chest was completely closed. His cough has been very slight, and the expectoration has not been more copious than before the accident.

The cure proceeded very favourably under the use of free depletion, and on the twentieth day he was so far recovered as to request to be allowed to leave the hospital. He was shortly after dismissed, (December 23d.)

Mr. Bell remarked, that the speedy and favourable termination of this case arose from the circumstance of the wound having taken on the adhesive process, and thus preventing the extension of inflammation through the cavity of the chest;* and that the frequent and effective blood-lettings, by preventing inflammation, must have greatly assisted in bringing about such a result.

* This subject is well illustrated by Mr. Hunter, in his chapter on Adhesion.

